# Editorial: The multiple applications of marine-derived bioactives

**DOI:** 10.3389/fphar.2025.1733987

**Published:** 2025-12-04

**Authors:** Patricia Rijo, Deniz Tasdemir, Ana Rotter

**Affiliations:** 1 CBIOS Lusófona’s Research Center for Biosciences and Health Technologies, Campo Grande, Lisbon, Portugal; 2 Research Institute for Medicines (iMed.ULisboa), Faculty of Pharmacy, Universidade de Lisboa, Lisbon, Portugal; 3 Centro de Química Estrutural, Institute of Molecular Sciences, Universidade de Lisboa, Campo Grande, Lisboa, Portugal; 4 Research Unit Marine Natural Product Chemistry, GEOMAR Helmholtz Centre for Ocean Research Kiel, Kiel, Germany; 5 Faculty of Mathematics and Natural Sciences, Kiel University, Kiel, Germany; 6 Marine Biology Station Piran, National Institute of Biology, Piran, Slovenia

**Keywords:** marine biotechnology, bioactivity, therapeutic, health, marine natural product

## Introduction

Marine biotechnology is a rapidly evolving field that focuses on the exploration and utilization of marine biological sources for development of products and services with societal benefits. The chemical constituents of marine organisms, especially secondary metabolites (i.e., marine natural products) that are designed for ecological functions in their ecosystems represent ideal candidates as drugs and other functional ingredients. However, despite the vast potential of marine biota, the ocean remains largely underexplored and undervalorized, presenting a significant opportunity for discovering alternative sources of drugs, agrochemicals, cosmeceuticals, nutraceuticals, bio-inspired materials and other industrially relevant chemicals (Rotter et al., 2021).

Marine biodiscovery is one of the main pillars of marine biotechnology and dedicated to unearthing of biologically active small molecules (Reddy et al., 2021). A rich catalogue of natural products mainly from marine invertebrates and algae has already contributed fundamentally to modern medicine, especially for the treatment of solid human cancers (Sigwart et al., 2021). Due to the limited supply issue of bioactive metabolites in sufficient quantities for drug development, microorganisms living symbiotically with marine macroorganisms, sediment or seawater emerge as sustainable and upscalable sources of marine bioactives (Rotter et al., 2021). However, the identification of the biodiscovery source is only the first step of the process. It normally proceeds with the appropriate extraction, biological profiling using diverse *in vitro* bioassays, chemical profiling/dereplication by untargeted metabolomics followed by isolation and characterization of the active compounds (Sabotič et al., 2024), and scaling up and production. Identification of the mechanism of pharmacological activity is also crucial for development of a marine natural product for medical applications. Hence, marine biodiscovery pipeline is inherently transdisciplinary and demands a long development cycle (up to 20 years or more), especially when developing new pharmaceuticals.

This Research Topic aims to provide an overview on the recent advances on marine biotechnology, addressing specific applications for the pharmaceutical and wellbeing industries. Although a limited number of manuscripts were accepted, they provide an interesting outlook on the current state of research in this field.

## Source of novel drug activities

There is increasing acceptance of alternative sources for marine natural products. While early marine biotechnology focused on macroorganisms—especially invertebrates such as sponges—their associated microbiomes, marine microorganisms from extreme or underexplored environments, and marine waste biomass are now gaining relevance. This shift is driven by growing concerns around sustainability, circular economy principles, and ethical considerations, and reflects the emerging potential of the blue bio-circular economy. This trend is mirrored in the manuscripts published within this Research Topic, where novel bioactivities are explored from marine waste biomass, such as fisheries processing by-products and beach wrack. One manuscript investigated seaweed collected from the intertidal rocky shore of northern Portugal and its seaweed-associated *Streptomyces* (Girão et al.). Two manuscripts addressed the circularity potential of marine biomass by targeting sardine scales and mackerel bones (Santos Filipe et al.; Wang et al.). Another manuscript presented a complementary approach—reusing previously isolated compounds—by screening a marine fungus-derived compound for new therapeutic effects (Rao et al.).

## Target natural products and their applications

To date, less than 20 marine-derived natural products have been approved as medicines by health authorities worldwide—such as the FDA in the United States, and equivalent agencies in the European Union, Australia—for the treatment of conditions including cancer, pain, and hypertriglyceridemia. However, many more marine-derived compounds are currently undergoing various stages of clinical and preclinical trials. Despite the relatively small number of approved drugs, the range of biological activities and potential applications of marine-derived organisms and their biomolecules is vastly broader. This is reflected in the diversity of target applications reported within this Research Topic, including anti-obesity treatment (Rao et al.), insomnia treatment using peptides (Wang et al.), antimicrobial and anticancer activities of prodigiosin (Girão et al.), and cosmetic and nutritional applications of collagen (Santos Filipe et al.).

## Pipeline of discovery

To gather further insights in the exploration and application of marine-derived bioactives, several steps are needed: isolation (when microorganisms are the target producers), and omics analyses, bioactivity screening, compound isolation, characterization, structure elucidation, animal experiments (using e.g., zebrafish or mice). Indeed, these steps were followed within the articles of this Research Topic.

## Future outlook

In summary, the marine biosynthetic potential for drug discovery still requires significant progress—particularly in structure prediction from gene sequences, biological activity forecasting from molecular structures, and the development of sustainable ‘green chemistry’ extraction methods (Almaliti and Gerwick, 2023). Looking ahead, scaling up production, addressing regulatory and standardization challenges, and integrating all pillars of sustainability—environmental, economic, and social—are essential. Some of these aspects must be considered even before research can be translated into clinical trials and real-world applications.
